# P113: The World Health Organization multimodal hand hygiene improvement strategy - a successful experience in a pediatric hospital

**DOI:** 10.1186/2047-2994-2-S1-P113

**Published:** 2013-06-20

**Authors:** AM Ribeiro, Francisca Luzilene Nogueira Della Guardia, Virginia Maria Ramos Sampaio, Rivânia Andrade Barros, Diana Maria da Silva, Maria Márcia de Sousa Cavalcante, Michely Pinto de Oliveira

**Affiliations:** 1 Control Infection, Hospital Infantil Albert Sabin, Fortaleza, Brazil

## Introduction

The impact of hand hygiene (HH) in the healthcare associated infection (HAI) control is already proven. Aiming toimprove adherence to HH, the Hospital Infantil Albert Sabin (HIAS) implemented the World Health Organization (WHO) multimodal hand hygiene improvement strategy since January 2009 and has developed an action plan for its sustainability for the next five years.

## Objectives

Evaluate the outcomes of implementation of the WHO strategy by analyzing rates of adherence to HH practice by health workers (HW) and HAI rates, in the period from 2009 to 2012.

## Methods

The strategy was implemented in three intensive care units (ICU) with 32 beds and 240 health workers of a Pediatric Brazilian Hospital. The protocol followed the WHO recommendations. Compliance to hand hygiene was monitored by direct observation.The first evaluation of hand hygiene was conducted in March 2009, before theintervention phase of the WHO strategy. The second evaluation was immediately after intervention phase, also in 2009. Two more evaluations were realized in 2010, three in 2011 and three in 2012. The HAI were identified by active search and the rates calculated according to Portaria 2616/98 Brazil – Ministry of Health. The program Epi Info 6 was used to analyze data**.**

## Results

The average annual rate of adherence to hand hygiene of three units for the years 2009, 2010, 2011 and 2012 were respectively: 50.8%, 73,8%, 78,8% and 85,8%. HAI incidence density in three ICUs before the implantation of the strategy (2008) was 21.3 infections per thousand patient/days. In 2009, 2010, 2011 and 2012 were respectively 17.4; 17.8; 12.6 and 12.2 infections per thousand patient/days.

## Conclusion

Increased adherence to HH practices during the study period and decreased density of nosocomial infection at the same time demonstrate the successful implementation of this strategy.

## Disclosure of interest

None declared

